# Morphology and Multigene Phylogeny Revealed Three New Species of *Helminthosporium* (*Massarinaceae*, *Pleosporales*) from China

**DOI:** 10.3390/jof9020280

**Published:** 2023-02-20

**Authors:** Ya-Fen Hu, Jing-Wen Liu, Zhao-Huan Xu, Rafael F. Castañeda-Ruíz, Kai Zhang, Jian Ma

**Affiliations:** 1 College of Agronomy, Jiangxi Agricultural University, Nanchang 330045, China; 2 Instituto de Investigaciones de Sanidad Vegetal, Calle 110 No. 514 e/5ta B y 5ta F, Playa, La Habana 11600, Cuba; 3 College of Forestry Engineering, Shandong Agriculture and Engineering University, Jinan 250100, China

**Keywords:** *Ascomycota*, asexual fungi, *Dothideomycetes*, multi-locus phylogeny, taxonomy

## Abstract

Saprobic hyphomycetes are highly diverse on plant debris. Over the course of our mycological surveys in southern China, three new *Helminthosporium* species, *H. guanshanense* sp. nov., *H. jiulianshanense* sp. nov. and *H. meilingense* sp. nov., collected on dead branches of unidentified plants, were introduced by morphological and molecular phylogenetic analyses. Multi-loci (ITS, LSU, SSU, *RPB2* and *TEF1*) phylogenetic analyses were performed using maximum-likelihood and Bayesian inference to infer their taxonomic positions within *Massarinaceae*. Both molecular analyses and morphological data supported *H. guanshanense*, *H. jiulianshanense* and *H. meilingense* as three independent taxa within *Helminthosporium*. A list of accepted *Helminthosporium* species with major morphological features, host information, locality and sequence data was provided. This work expands our understanding of the diversity of *Helminthosporium*-like taxa in Jiangxi Province, China.

## 1. Introduction

Hyphomycetes, a group of anamorphic *Ascomycota*, are highly diverse in aquatic and terrestrial habitats and distributed worldwide on many natural substrates such as plant tissues, wood and bark, dung, insects and other arthropods and other fungi including lichens [1]. More than 30,000 species of asexual fungi are recorded worldwide, with 2500 hyphomyceteous genera [2,3]. The most comprehensive occurrence of this group is in the northern temperate regions, with little recorded in tropical and subtropical areas [1,3]. China is considered an important reservoir of biodiversity by the Convention on Biological Diversity. However, more research on fungal diversity in China is needed.

*Helminthosporium* is a hyphomyceteous genus in the family *Massarinaceae* of the order *Pleosporales*, which was established by Link [4] and typified by *H. velutinum* Link. It is an old, species-rich genus, and its taxonomic history is complex. To date, more than 770 epithets for *Helminthosporium* are listed in Index Fungorum [3], but most *Helminthosporium* species are not congeneric with the generic type in development of conidia and conidiophores and were excluded from *Helminthosporium* [5,6,7,8,9,10,11,12,13]. Ellis [7] synonymised numerous species with *H. velutinum*, and accepted 10 *Helminthosporium* species based on extensive morphological investigations. Siboe et al. [10] accepted 27 *Helminthosporium* species, and provided a synopsis table summarizing their main diagnostic morphological characters. Since then, 45 further species have been added to the genus [13,14,15,16,17,18,19,20,21,22,23,24,25,26,27,28,29,30,31]. However, nine species, viz., *H. apicale* V. Rao and de Hoog, *H. bigenum* Matsush., *H. catenatum* Matsush., *H. dictyoseptatum* S. Hughes, *H. hypselodelphyos* M.B. Ellis, *H. parvum* R.F. Castañeda and W.B. Kendr., *H. senseletii* Bhat and B. Sutton, *H. varium* Alves-Barb., Malosso and R.F. Castañeda and *H. zombaense* B. Sutton, were excluded [11,12,13]. Two species, *H. cylindrosporum* Matsush. [32] and *H. gigasporum* Shirouzu and Y. Harada [33], are, respectively, synonymised with *H. matsushimae* D.W. Li, K. Zhang and R.F. Castañeda [13] and *H. magnisporum* Shirouzu and Y. Harada [17] because they are a later homonym of *H. cylindrosporum* Sacc. and *H. gigasporum* Berk. and Broome, respectively. *Helminthosporium dimorphosporum* Hol.-Jech. is regarded as a questionable species that produces distoseptate and euseptate conidia and does not fit the *Helminthosporium* generic concept [13]. Thus, following Siboe et al.’s [10] treatment, the genus currently comprises 60 species.

Most *Helminthosporium* species are described based on their anamorph alone, and only six species, *H. massarinum* Kaz. Tanaka, K. Hiray. and Shirouzu, *H. microsorum* D. Sacc., *H. oligosporum* (Corda) S. Hughes, *H. quercicola* (M.E. Barr) Voglmayr and Jaklitsch, *H. quercinum* Voglmayr and Jaklitsch and *H. tiliae* (Link) Fr., have been linked with *Massaria*- or *Splachnonema*-like teleomorphs, of which five have been confirmed by pure culture and sequence data [22,24]. Recent molecular data demonstrated that *Helminthosporium* is a polyphyletic genus [30,34], with some members mixed with other taxa of *Byssothecium*, *Haplohelminthosporium*, *Helminthosporiella*, *Pseudosplanchnonema* and *Synhelminthosporium* [30,34].

Jiangxi, located in the southeast of China, is one of the most biodiverse provinces. Its preserved superior ecological environment, humid subtropical climate and abundant plant resources would suggest that the province also has great fungal diversity. During a survey of saprobic hyphomycetes from plant debris in this province, three interesting hyphomycetes belonging to the genus *Helminthosporium* (*Massarinaceae*, *Pleosporales*) were collected on dead branches. Based on the multi-locus phylogenetic analysis and morphological examination, they are introduced as new to science in the present study.

## 2. Materials and Methods

### 2.1. Sample Collection, Isolation and Morphology

Samples of dead branches were collected from humid environments and river banks in the subtropical forests of Jiangxi Province, China, and placed in Ziploc™ plastic bags. Samples were processed and examined following the methods described in Ma et al. [35]. Colonies on decaying wood surfaces were examined and visually observed with a stereomicroscope (Motic SMZ-168, Xiamen, China) from low (0.75 times) to high (5 times) magnification. Fresh colonies were picked with sterile needles at a stereomicroscope magnification of 5 times, placed on a slide with a drop of lactic acid–phenol solution (lactic acid, phenol, glycerin, sterile water; 1:1:2:1, respectively), then placed under an Olympus BX 53 light microscope fitted with an Olympus DP 27 digital camera (Olympus Optical Co., Tokyo, Japan) for microscopic morphological characterization. The tip of a sterile toothpick dipped in sterile water was used to capture the conidia of the target colony directly from the specimen; the conidia were then streaked on the surface of potato dextrose agar (PDA; 20% potato + 2% dextrose + 2% agar, *w/v*) and incubated in an incubator at 25 °C overnight. The single germinated conidia were transferred to fresh PDA plates following the method of Goh [36] and incubated in an incubator at 25 °C. Culture characteristics were examined and recorded after 3 days and later at regular intervals for 3 days. Colony colors were assessed according to the charts of Rayner [37]. All fungal strains were stored in 10% sterilized glycerin at 4 °C for further studies. The studied specimens and cultures were deposited in the Herbarium of Jiangxi Agricultural University, Plant Pathology, Nanchang, China (HJAUP). The names of the new taxa were registered in Index Fungorum [3].

### 2.2. DNA Extraction, PCR Amplification and Sequencing

Genomic DNA was extracted from fungal mycelia grown on PDA, using the Solarbio Fungi Genomic DNA Extraction Kit (Solarbio, Beijing, China) following the manufacturer’s protocol. DNA amplification was performed by polymerase chain reaction (PCR) using the respective loci (ITS, LSU, SSU, *TEF1* and *RPB2*). The following primer sets were used for these genes: ITS: ITS5/ITS4 [38]; LSU: 28S1-F/28S3-R [39]; SSU: 18S-F/18S-R [39]; *TEF1*: EF1-983F/EF1-2218R [28,40] and *RPB2*: RPB2-5F2 [41]/fRPB2-7cR [42]. The amplifications were performed in a 25 μL reaction volume containing 12.5 μL of 2 × Power Taq PCR MasterMix, 1 μL of each forward and reverse primer, 1 μL of DNA template and 9.5 μL of ddH_2_O. The PCR thermal cycle program for ITS, LSU, SSU and *TEF1* amplification was as follows: 95 °C for 3 min, followed by 35 cycles of denaturation at 94 °C for 15 s, annealing at 55 °C for 15 s, elongation at 72 °C for 30 s and finally extended at 72 °C for 5 min. Regions of *RPB2* were amplified with annealing at 59 °C for 15 s, elongation at 72 °C for 2 min and others consistent with the above procedure. The PCR products were checked on 1% agarose gel electrophoresis stained with ethidium bromide. Purification and sequencing of PCR products were carried out by Beijing Tsingke Biotechnology Co., Ltd. China. New sequences generated in this study were deposited in the NCBI GenBank (www.ncbi.nlm.nih.gov, accessed on 5 January 2023; Table 1).

### 2.3. Phylogenetic Analyses

The newly generated sequences together with other sequences obtained from GenBank (Table 1) were aligned using MAFFTv.7 [43] on the online server (http://maffTh.cbrc.jp/alignment/server/, accessed on 5 January 2023), and optimized manually when needed. Phylogenetic analyses were conducted individually for each locus at first and then for a combined dataset of five gene loci (ITS, LSU, SSU, *TEF1* and *RPB2*). The tandem sequences of ITS, SSU, LSU, *TEF1* and *RPB2* were obtained by Phylosuite software v1.2.1 [44] under “Concatenate Sequence”, and absent sequence data in the alignments were treated with a question mark as missing data. The best-fitting nucleotide substitution models for each alignment dataset were selected using ModelFinder [45]. Maximum-likelihood (ML) and Bayesian inference (BI) were used to analyze the dataset after splicing. Maximum-likelihood phylogenies were inferred using IQ-TREE [46] under an Edge-linked partition model for 10,000 ultrafastbootstraps [47]. The optimal ML tree search was conducted with 1000 separate runs using the default algorithm of the program from a random starting tree for each run. The best-fit model was TIM3 + F + R3 for *TEF1*, TIM2e + I + G4 for ITS, TNe + R4 for LSU, TN + F + I + G4 for *RPB2* and K2P + R2 for SSU. Bayesian inference phylogenies were inferred using MrBayes 3.2.6 [48] under a partition model (2 parallel runs, 2,000,000 generations), in which the initial 25% of sampled data were discarded as burn-in. The best-fit model was GTR + F + I + G4 for *RPB2*, ITS and LSU, GTR + F + G4 for *TEF1* and HKY + F + G4 for SSU. ModelFinder [45] was used to select the best-fit partition model (Edge-linked) using BIC criterion. These trees were visualized using FigTree v. 1.4.4 (http://tree.bio.ed.ac.uk/software/figtree, accessed on 16 January 2023), with editing and typesetting using Adobe Illustrator CS v. 5.

## 3. Results

### 3.1. Molecular Phylogeny

**Figure 1:**
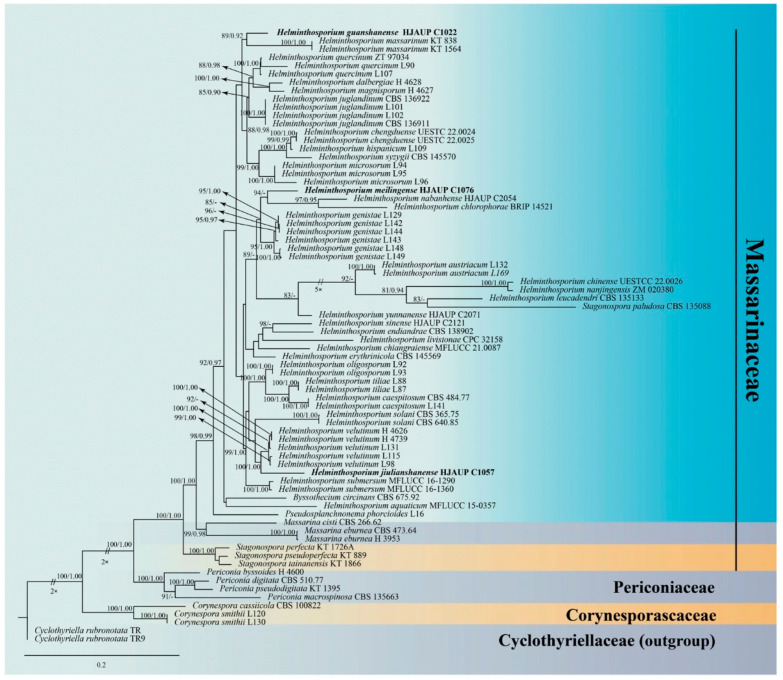
Phylogram generated from maximum-likelihood analysis based on combined ITS, LSU, SSU, *TEF1* and *RPB2* sequence data for the families *Corynesporaceae*, *Massarinaceae* and *Periconiaceae*. The ML and BI bootstrap support values above 75% and 0.90 are given above the nodes. The tree is rooted to *Cyclothyriella rubronotata* (TR) and *C. rubronotata* (TR9). Strains from the current study are in bold. Some branches were shortened according to the indicated multipliers.

In this study, five gene regions of ITS, LSU, SSU, *TEF1* and *RPB2* were obtained successfully except for *H. jiulianshanense,* which lack SSU and *TEF1* sequences. Phylogenetic relationships of three *Helminthosporium* species were assessed in the combined analysis using 5 gene regions of 74 strains representing 48 species in *Massarinaceae* and related families (*Periconiaceae*, *Corynesporascaceae* and *Cyclothyriellaceae*). The combined dataset (*TEF1*:1-372, ITS:1373-2042, LSU:2043-2937, *RPB2*:2938-4068, SSU:4069-5129) was composed of 2274 distinct patterns, 1900 parsimony-informative, 319 singleton sites and 2910 constant sites. A total of 5 single-locus datasets, ITS, LSU, SSU, *RPB2* and *TEF1,* contained 670, 895, 1061, 1131 and 1372 parsimony informative sites, respectively. *Cyclothyriella rubronotata* (TR) and *C. rubronotata* (TR9) served as outgroup taxa. Maximum-likelihood and Bayesian inference analyses of the combined datasets resulted in phylogenetic reconstructions with largely similar topologies, and bootstrap support values for maximum-likelihood higher than 75% and Bayesian posterior probabilities greater than 0.90 are given above the nodes. The best-scoring ML consensus tree (lnL = −38,302.006) with ultrafast bootstrap values from ML analyses and posterior probabilities from MrBayes analysis at the nodes are shown in Figure 1. Our newly obtained *Helminthosporium* isolates represent three different clades. The strain of *H. guanshanense* (HJAUP C1022) forms a distinct clade sister to two different strains of *H*. *massarinum* (KT 838 and KT 1564.7) with 89%ML/0.92BI bootstrap support; *H. jiulianshanense* (HJAUP C1057) forms a high-support clade (100%ML/1.00BI) with the lineage consisting of five different strains of *H. velutinum* (H4626, H4739, L131, L115 and L98); *H. meilingense* (HJAUP C1076) clustered as a sister taxon to the clade containing *H*. *nabanhense* (HJAUP C2054) and *H. chlorophorae* (BRIP 14521) with 94%ML/0.68BI bootstrap support.

### 3.2. Taxonomy

**Figure 2:**
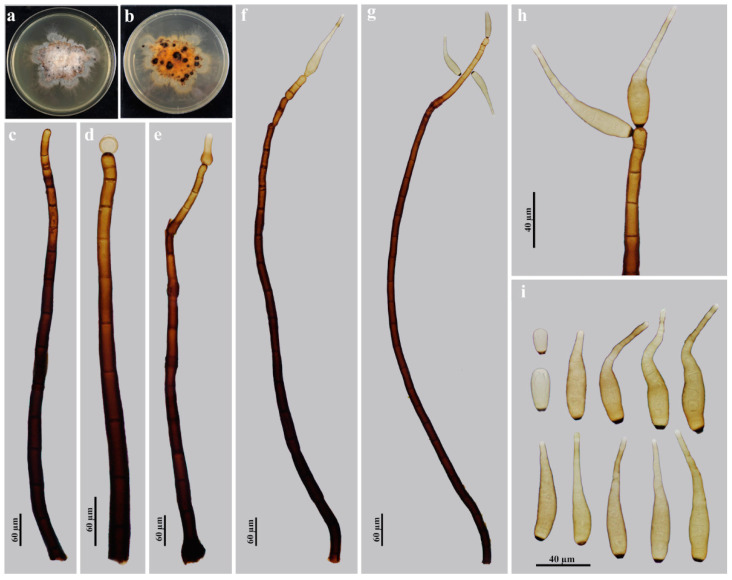
*Helminthosporium guanshanense* (HJAUP M1022, holotype): (**a**) surface of colony after 4 weeks on PDA; (**b**) reverse of colony after 4 weeks on PDA; (**c**) conidiophore and conidiogenous cells; (**d**–**g**) conidiophores, conidiogenous cells and conidia; (**h**) conidiogenous cells and conidia; (**i**) conidia.

*Helminthosporium guanshanense* Y.F. Hu and Jian Ma, sp. nov., Figure 2.

Indexfungorum number: IF900239.

Etymology: The name refers to Guanshan Nature Reserve, the locality where the fungus was collected.

Holotype: HJAUP M1022.

Description: Saprobic on dead branches in terrestrial habitats. Anamorph hyphomycetous. *Colonies* on natural substrate effuse, scattered, hairy, brown to dark brown. *Mycelium* superficial and immersed in the substratum, composed of branched, septate, smooth, pale brown to brown, thick-walled hyphae. *Conidiophores* macronematous, mononematous, solitary or in groups of 2–4, simple, erect, straight or flexuous, cylindrical, smooth, 10–34-septate, blackish brown, paler towards the apex, sometimes with cylindrical, enteroblastic percurrent extensions with well-defined small pores at the apex and laterally beneath the upper 1–5 septa, 282.5–977.5 × 15–20 μm ($\overset{¯}{x}$ = 700.3 × 16 μm, *n* = 10). *Conidiogenous cells* polytretic, integrated, terminal and intercalary, cylindrical, brown, smooth. Conidial secession schizolytic. *Conidia* acropleurogenous, solitary, dry, obclavate, rostrate, straight or curved, 5–14-distoseptate, pale brown, smooth, 55–107.5 × 10–15 µm ($\overset{¯}{x}$ = 85.8 × 13.7 μm, *n* = 21), tapering to 4.5–7.5 µm near the apex, 5–9 μm wide at the base.

Culture characteristics: Colony on PDA reaching 55–65 mm diam. after 4 weeks in an incubator under dark conditions at 25 °C, irregular circular, surface reddish dark brown with gray white in the center and gray mat of aerial hyphae at the margin; reverse rosy-brown with black dots and pale brown periphery.

Material examined: China, Jiangxi Province, Yichun City, the Guanshan National Nature Reserve, on dead branches of an unidentified broadleaf tree, 25 June 2021, Y.F. Hu (HJAUP M1022, *holotype*; ex-type culture permanently preserved in a metabolically inactive state by freezing HJAUP C1022).

Notes: The phylogenetic tree shows that the strain of *H. guanshanense* (HJAUP C1022) clusters with the ex-type strain of *H. massarinum* (KT 838 and KT 1564^T^). The BLASTn analysis of *H. guanshanense* (HJAUP C1022) and *H. massarinum* (KT 1564 ^T^) shows 94% identity (432/461, 2 gaps) using ITS, 99% identity (570/578, 2 gaps) using LSU, 99% identity (447/448, no gap) using SSU and 95% identity (865/910, no gap) using *RPB2*. Moreover, *H. guanshanense* differs from *H. massarinum* [22] by its wider conidiophores (15–20 μm vs. 7–9 μm) and longer conidia (55–107.5 × 10–15 µm vs. 17–56.5 × 5–9 μm) with more distosepta (5–14 vs. 1–8). *Helminthosporium guanshanense* also superficially resembles *H. quercinum* [24], but the latter has smaller conidiophores [(40–)74–199(–332) × 11–18 µm vs. 282.5–977.5 × 15–20 μm], and longer conidia [(47–)78–130(–201) × (13.2–)15.3–18.0(–20.5) µm vs. 55–107.5 × 10–15 µm] with 8–13(–20) distosepta.

**Figure 3:**
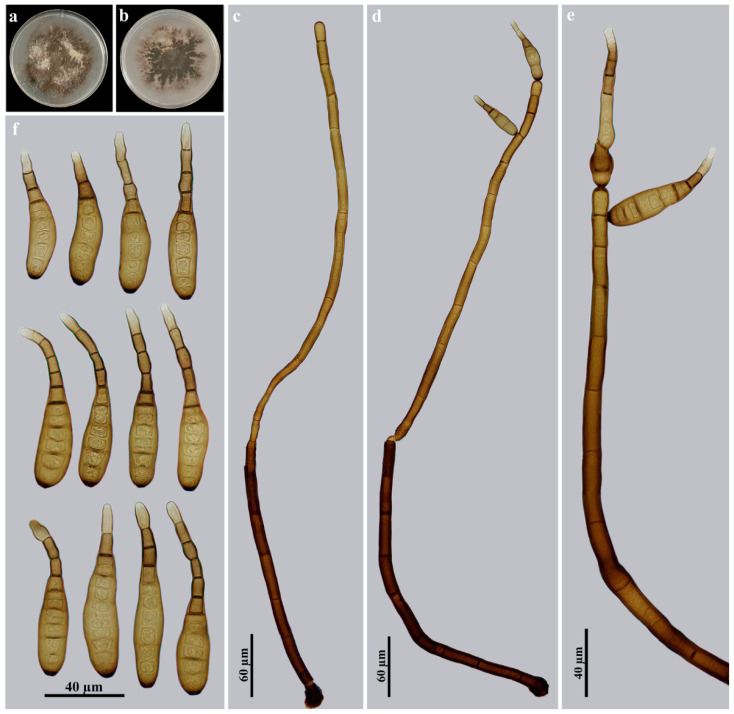
*Helminthosporium jiulianshanense* (HJAUP M1057, holotype): (**a**) surface of colony after 4 weeks on PDA; (**b**) reverse of colony after 4 weeks on PDA; (**c**) conidiophore and conidiogenous cells; (**d**,**e**) conidiophores, conidiogenous cells and conidia; (**f**) conidia.

*Helminthosporium jiulianshanense* Y.F. Hu and Jian Ma, sp. nov., Figure 3.

Index Fungorum number: IF900240.

Etymology: The name refers to Jiulianshan National Forest Park, the locality where the fungus was collected.

Holotype: HJAUP M1057.

Description: Saprobic on decaying wood in terrestrial habitats. Anamorph hyphomycetous. *Colonies* on natural substrate effuse, scattered, hairy, brown to dark brown. *Mycelium* superficial and immersed in the substratum, composed of branched, septate, smooth, pale brown to brown, thick-walled hyphae. *Conidiophores* macronematous, mononematous, solitary or in groups of 2–3, simple, erect, straight or flexuous, cylindrical, smooth, 10–21-septate, blackish brown, paler towards the apex, with one cylindrical, enteroblastic percurrent extension with well-defined small pores at the apex and laterally beneath the upper 1–4 septa, (290–)531–712 × 10–15 μm ($\overset{¯}{x}$ = 520 × 13 µm, *n* = 10). *Conidiogenous cells* polytretic, integrated, terminal and intercalary, cylindrical, brown, smooth. Conidial secession schizolytic. *Conidia* acropleurogenous, solitary, dry, obclavate, rostrate, straight or curved, 6–13-distoseptate, pale brown to brown, smooth, (57–)78–120 × 13–17.5 µm ($\overset{¯}{x}$ = 93 × 14.8 µm, *n* = 20), tapering to 4–6.5 µm near the apex, 5–9 μm wide at the base.

Culture characteristics: Colony on PDA reaching 70–78 mm diam. after 4 weeks in an incubator under dark conditions at 25 °C, irregular circular, surface velvety, with dense, dark brown mycelium plus white patches of aerial hyphae, and becoming sparser towards the edge; reverse gray with sparser black patches in the center.

Material examined: China, Jiangxi Province, Ganzhou City, Jiulianshan National Forest Park, on dead branches of an unidentified broadleaf tree, 26 June 2021, Y.F. Hu (HJAUP M1057, *holotype*; ex-type culture permanently preserved in a metabolically inactive state by freezing HJAUP C1057).

Notes: The phylogenetic tree shows that the strain of *H. jiulianshanense* (HJAUP C1057) clusters with five different strains of *H. velutinum* (H4626, H4739, L131^T^, L115, L98), and they form a sister clade to *H. solani* (CBS 365.75 and CBS640.85). The BLASTn analysis of *H. jiulianshanense* (HJAUP C1057) and *H. velutinum* (L131^T^) shows 96% identity (563/585, 6 gaps) using ITS, 98% identity (574/583, 4 gaps) using LSU and 94% identity (880/934, 7 gaps) using *RPB2*. Moreover, *H. jiulianshanense* morphologically differs from *H. velutinum* [24] in the size of the conidiophores [(290–)531–712 × 10–15 μm vs. (163–)340–698(–960) × 14–26 µm] and conidia [(57–)78–120 × 13–17.5 µm vs. (42–)56–89(–142) × (11–)14.3–18.5(–24.7) µm] and conidial distosepta (6–13 vs. 6–8). In addition, *H. jiulianshanense* morphologically differs from *H. solani* Durieu and Mont. [7,49] by its longer conidiophores [(290–)531–712 μm vs. 120–600 μm] and longer conidia [(57–)78–120 µm vs. 24–85 µm] with more distosepta (6–13 vs. 2–8).

**Figure 4:**
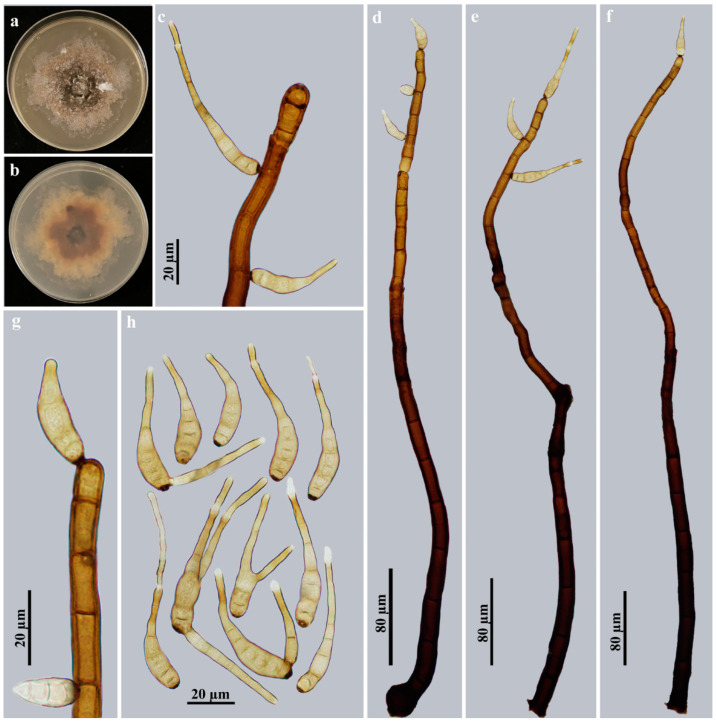
*Helminthosporium meilingense* (HJAUP M1076, holotype): (**a**) surface of colony after 4 weeks on PDA; (**b**) reverse of colony after 4 weeks on PDA; (**c,g**) conidiogenous cells and conidia; (**d**–**f**) conidiophores, conidiogenous cells and conidia; (**h**) conidia.

*Helminthosporium meilingense* Y.F. Hu and Jian Ma, sp. nov., Figure 4.

Index Fungorum number: IF900241.

Etymology: The name refers to Meiling Scenic Spot, the locality where the fungus was collected.

Holotype: HJAUP M1076.

Description: Saprobic on decaying wood in terrestrial habitats. Anamorph hyphomycetous. *Colonies* on natural substrate effuse, scattered, hairy, brown to dark brown, velvety. *Mycelium* superficial and immersed in the substratum, composed of branched, septate, smooth, pale brown to brown, thick-walled hyphae. *Conidiophores* macronematous, mononematous, solitary or in groups of 2–4, simple, erect, straight or flexuous, cylindrical, smooth, 18–29-septate, blackish brown, paler towards the apex, with several cylindrical, enteroblastic percurrent extensions with well-defined small pores at the apex and laterally beneath the upper 1–5 septa, 544–712.5 × 12.5–17 μm ($\overset{¯}{x}$ = 622 × 15.2 μm, *n* = 8). *Conidiogenous cells* polytretic, integrated, terminal and intercalary, cylindrical, brown, smooth, with noncicatrized, distinct pores. Conidial secession schizolytic. *Conidia* acropleurogenous, solitary, dry, obclavate, rostrate, straight or curved, 6–13-distoseptate, pale brown, smooth, (20.7–)41.5–82.8 × 6.9–10.4 µm ($\overset{¯}{x}$ = 64 × 8 µm, *n* = 29), tapering to 1.7–3.5 µm near the apex, 3–7 μm wide at the base. Basal cell or apical portion sometimes with branches that developed a rostrate.

Culture characteristics: Colony on PDA reaching 60–70 mm diam. after 4 weeks in an incubator under dark conditions at 25 °C, irregular circular, surface gray-brown with blackish brown in the center and gray mat of aerial hyphae at the margin; reverse rosy-brown with dark brown center and pale brown periphery.

Material examined: China, Jiangxi Province, Nanchang City, Meiling Scenic Spot, on dead branches of an unidentified broadleaf tree, 27 June 2021, Y.F. Hu (HJAUP M1076, *holotype*; ex-type culture permanently preserved in a metabolically inactive state by freezing HJAUP C1076).

Notes: The phylogenetic tree shows that the strain of *H. meilingense* (HJAUP C1076) forms an independent clade and clusters with the strains of *H. nabanhense* (HJAUP C2054) and *H. chlorophorae* (BRIP 14521). The BLASTn analysis of *H. meilingense* (HJAUP C1076) and *H. nabanhense* (HJAUP C2054) shows 92% identity (450/487, 4 gaps) using ITS, 96% identity (562/583, 7 gaps) using LSU, 99% identity (883/86, 2 gaps) using SSU and 94% identity (712/761, 1 gap) using *TEF1*; of *H. meilingense* (HJAUP C1076) and *H. chlorophorae* (BRIP 14521) show 90% identity (427/473, 12 gaps) using ITS. Moreover, *H. meilingense* is significantly different from *H. nabanhense* Jing W. Liu and Jian Ma [31] in its longer conidiophores (544–712.5 × 12.5–17 μm vs. 365–557 × 6.5–13.5 μm) and longer conidia [(20.7–)41.5–82.8 μm vs. 26.5–46.5 μm] with more distosepta (6–13 vs. 3–6) and from *H. chlorophorae* M.B. Ellis [7] in its longer conidiophores (544–712.5 × 12.5–17 μm vs. 120–270 × 7–10 μm) and smaller conidia [(20.7–)41.5–82.8 × 6.9–10.4 μm vs. 52–102 × 8–11 μm] with more distosepta (6–13 vs. 6–9). In addition, *H. meilingense* further differs from *H. nabanhense* and *H. chlorophorae* in producing simple or branched conidia.

## 4. Discussion

The establishment of *Helminthosporium* was based on morphological studies. More than 770 epithets for *Helminthosporium* have been listed in Index Fungorum [3]. Members in the genus mainly occur in the asexual morph, usually forming effuse, hairy colonies on decaying leaf or twig litter. The generic concept of *Helminthosporium* is based on the characteristics of asexual morph and is mainly characterized by distinct, determinate or percurrently extending conidiophores with a well-defined small pore at the apex and/or laterally beneath the septa and tretic, integrated, terminal or intercalary conidiogenous cells that produce solitary (rarely in short chains), clavate or obclavate, distoseptate conidia usually with a distinct dark brown to black scar at the base [1,4,7,11,24,50,51]. Voglmayr and Jaklitsch [24] transferred four *Corynespora* species to *Helminthosporium* based on molecular phylogenetic analyses, which led to the characters delineating the genus *Helminthosporium* also covering the criteria of *Corynespora*. The traditional distinction between monotretic vs. polytretic conidiogenous cells for separating *Corynespora* and *Helminthosporium* is shown to be insignificant in a phylogenetic context.

The taxonomic history of the genus *Helminthosporium* is complex. Many graminicolous taxa conventionally named as “*Helminthosporium*” species have been reclassified into the genera *Bipolaris*, *Curvularia*, *Drechslera* and *Exserohilum* [8], and several lignicolous species were recently transferred to *Ellismarsporium*, *Mirohelminthosporium, Stanhughesiella*, *Varioseptispora* and other genera due to their atypical features in *Helminthosporium* [11,12,13]. Konta et al. [34] listed 216 *Helminthosporium* species based on records from Species Fungorum, but many species are identified based only on morphological studies, and only 33 species have sequence data so far. Morphological comparison is important for fungal identification, but species identification only based on morphological studies is not comprehensive [31]. There is presently a strong tendency to evaluate previous described *Helminthosporium* species by molecular methods. Thus, resurrection of the genus *Helminthosporium* and studying their diversity and biology by a morpho-molecular approach are urgently necessary, which may be helpful to clarify the taxonomic status of many doubtful species and some important plant pathogens [23,28].

The genus *Helminthosporium* has a worldwide distribution with species recorded from a wide range of hosts [23,24,30,34,51,52,53,54,55]. However, the number of *Helminthosporium* species is very confusing in the recent monograph [24,28,56,57,58]. For example, Kirk et al. [56] recorded in the Dictionary of the Fungi that the genus comprises c. 35 species. Wijayawardene et al. [57,58] respectively estimated the genus including c. 40 and 416 species. Voglmayr and Jaklitsch [24] approximated the number of taxa accepted in *Helminthosporium* is about 46. In addition, Voglmayr and Jaklitsch [24] synonymised *Exosporium* with *Helminthosporium* and evaluated 17 *Helminthosporium* species by morphological and molecular systematic analysis. Konta et al. [34] listed 216 *Helminthosporium* species based on the records of Species Fungorum 2021. Considering that the number of *Helminthosporium* species does not match and many subsequent authors followed Siboe et al.’s [10] treatment, 63 species are currently accommodated in this genus. A checklist for these 63 *Helminthosporium* species, including major morphological features, host information, locality and sequence data, is provided in Table 2. Most of these are commonly collected from leaves and decaying wood in terrestrial habitats [22,24,30,34], and only two species, *H. aquaticum* Hong Y. Su, Z.L. Luo and K.D. Hyde and *H. submersum* Z.L. Luo, N. Zhao, K.D. Hyde and H.Y. Su, are recorded in freshwater habitats [23,28]. Thus, large-scale surveys of fungal resources in aquatic and terrestrial habitats with different geographic regions, ecological environment, vegetation type and climatic conditions will contribute to the knowledge of the fungal diversity and to a better understanding of the doubtful species, further clarifying their taxonomic status by phylogenetic analyses.

## Figures and Tables

**Table 1:** Species and GenBank accession numbers of DNA sequences used in this study. New sequences are in bold.

Species	Culture/Specimen No.	GenBank Accession Numbers				
		SSU	LSU	ITS	*RPB2*	*TEF1*
*Byssothecium circinans*	CBS 675.92	GU205235	GU205217	OM337536	DQ767646	GU349061
*Corynespora cassiicola*	CBS 100822	GU296144	GU301808	–	GU371742	GU349052
*C. smithii*	L120	–	KY984297	KY984297	KY984361	KY984435
*C. smithii*	L130	KY984419	KY984298	KY984298	KY984362	KY984436
*Cyclothyriella rubronotata*	TR = CBS 121892	–	KX650541	KX650541	KX650571	KX650516
*C. rubronotata*	TR9 ^ET^ = CBS 141486	KX650507	KX650544	KX650544	KX650574	KX650519
*Helminthosporium aquaticum*	S-096 ^HT^ = MFLUCC 15-0357	KU697310	KU697306	KU697302	–	–
*H. austriacum*	L132 ^HT^ = CBS 139924	KY984420	KY984301	KY984301	KY984365	KY984437
*H. austriacum*	L169 = CBS 142388	–	KY984303	KY984303	KY984367	KY984439
*H. caespitosum*	L99 ^ET^ = CBS 484.77	KY984421	JQ044448	JQ044429	KY984370	KY984440
*H. caespitosum*	L141	–	KY984305	KY984305	KY984368	–
*H. chengduense*	UESTC 22.0024 = CGMCC 3.23575 ^HT^	ON557757	ON557745	ON557751	ON563073	ON600598
*H. chengduense*	UESTC 22.0025	ON557756	ON557744	ON557750	ON563072	ON600597
*H. chiangraiense*	MFLUCC 21-0087 ^HT^	–	MZ538538	MZ538504	–	–
*H. chinense*	UESTCC 22.0026 = CGMCC 3.23570 ^HT^	ON557760	ON557748	ON557754	–	ON600601
*H. chlorophorae*	BRIP 14521	–	–	AF120259	–	–
*H. dalbergiae*	H4628 = MAFF 243853	AB797231	AB807521	LC014555	–	AB808497
*H. endiandrae*	CBS 138902 = CPC 22194 ^HT^	–	KP004478	KP004450	–	–
*H. erythrinicola*	CBS 145569 ^HT^ = CPC 35291	–	MK876432	NR_165563	MK876486	–
*H. genistae*	L129 = CBS 139922	KY984423	KY984309	KY984309	KY984373	–
*H. genistae*	L142 ^ET^ = CBS142597	–	KY984310	KY984310	KY984374	–
*H. genistae*	L143 = CBS 139927	–	KY984311	KY984311	KY984375	–
*H. genistae*	L144 = CBS 139928	–	KY984312	KY984312	KY984376	–
*H. genistae*	L148 = CBS 139929	–	KY984315	KY984315	KY984379	–
*H. genistae*	L149 = CBS 139930	–	KY984316	KY984316	KY984380	–
***H. guanshanense***	**HJAUP C1022 ^ET^**	**OQ172247**	**OQ172239**	**OQ172249**	**OQ234978**	**OQ256247**
*H. hispanicum*	L109 ^HT^ = CBS 136917	KY984424	KY984318	KY984318	KY984381	KY984441
***H. jiulianshanense***	**HJAUP C1057 ^ET^**	**–**	**OQ172253**	**OQ172245**	**OQ234979**	**–**
*H. juglandinum*	L101 = CBS 136912	–	KY984319	KY984319	KY984382	KY984442
*H. juglandinum*	L102 = CBS 136913	–	KY984320	KY984320	–	–
*H. juglandinum*	L118 ^HT^ = CBS 136922	–	KY984321	KY984321	KY984383	KY984443
*H. juglandinum*	L97 = CBS 136911	KY984425	KY984322	KY984322	–	–
*H. leucadendri*	CBS 135133 = CPC19345 ^HT^	–	KF251654	KF251150	KF252159	KF253110
*H. livistonae*	CPC 32158 = CBS144413 ^HT^	–	NG_064539	NR_160348	–	–
*H. magnisporum*	H4627 = MAFF 239278 = TS 33 ^HT^	AB797232	AB807522	AB811452	–	AB808498
*H. massarinum*	KT 838 ^ET^ = MAFF239604	AB797233	AB807523	AB809628	–	AB808499
*H. massarinum*	KT 1564 ^HT^ = MAFF 239605 = CBS 139690	AB797234	AB807524	AB809629	–	AB808500
***H. meilingense***	**HJAUP C1076 ^ET^**	**OQ172246**	**OQ172238**	**OQ172244**	**OQ234980**	**OQ234981**
*H. microsorum*	L94	KY984426	KY984327	KY984327	KY984388	KY984446
*H. microsorum*	L95	–	KY984328	KY984328	KY984389	KY984447
*H. microsorum*	L96 ^ET^ = CBS 136910	KY984427	KY984329	KY984329	KY984390	KY984448
*H. nabanhense*	HJAUP C2054 ^ET^	OP555400	OP555398	OP555394	–	OP961931
*H. nanjingense*	ZM020380 = HHAUF020380	–	–	KF192322	–	–
*H. oligosporum*	L92 = CBS 136908	KY984428	KY984332	KY984332	KY984393	KY984450
*H. oligosporum*	L93 ^ET^ = CBS 136909	–	KY984333	KY984333	KY984394	KY984451
*H. quercinum*	ZT-97034 = CBS 112393	–	KY984334	KY984334	KY984395	KY984452
*H. quercinum*	L107 = CBS 136915	–	KY984336	KY984336	KY984397	–
*H. quercinum*	L90 ^HT^ = CBS 136921	KY984429	KY984339	KY984339	KY984400	KY984453
*H. sinense*	HJAUP C2121 ^ET^	OP555399	OP555397	OP555393	–	OP961932
*H. solani*	CBS 365.75	KY984430	KY984341	KY984341	KY984402	KY984455
*H. solani*	CBS 640.85	–	KY984342	KY984342	KY984403	–
*H. submersum*	MFLUCC 16-1360 ^HT^	MG098796	MG098787	–	–	MG098586
*H. submersum*	MFLUCC 16-1290 ^PT^	MG098797	MG098788	MG098780	MG098592	MG098587
*H. syzygii*	CPC35312 = CBS 145570 ^HT^	–	MK876433	NR_165564	MK876487	–
*H. tiliae*	L87 = CBS 136906	–	KY984344	KY984344	KY984405	–
*H. tiliae*	L88 ^ET^ = CBS 136907	KY984431	KY984345	KY984345	KY984406	KY984457
*H. velutinum*	H4626	AB797240	AB807530	LC014556	–	AB808505
*H. velutinum*	H4739	AB797235	AB807525	LC014557	–	AB808501
*H. velutinum*	L115 = CBS 136924	–	KY984347	KY984347	KY984408	KY984458
*H. velutinum*	L131 ^ET^ = CBS 139923	KY984432	KY984352	KY984352	KY984413	KY984463
*H. velutinum*	L98	KY984433	KY984359	KY984359	KY984417	KY984466
*H. yunnanense*	HJAUP C2071 ^ET^	OP555392	OP555396	OP555395	OP961934	OP961933
*Massarina cisti*	CBS 266.62 = JCM 14140 ^HT^	AB797249	AB807539	LC014568	–	AB808514
*M. eburnea*	CBS 473.64	AF164367	GU301840	AF383959	GU371732	GU349040
*M. eburnea*	H3953 = CBS 139697	AB521718	AB521735	LC014569	–	AB808517
*Periconia byssoides*	H4600 = MAFF243872	AB797280	AB807570	LC014581	–	AB808546
*P. digitata*	CBS 510.77	AB797271	AB807561	LC014584	–	AB808537
*P. macrospinosa*	CBS 135663	KP184080	KP184038	KP183999	–	–
*P. pseudodigitata*	KT 1395 = CBS139699 = MAFF 239,676 ^HT^	NG_064850	NG_059396	NR_153490	–	AB808540
*Pseudosplanchnonema phorcioides*	L16 = CBS 122935	KY984434	KY984360	KY984360	KY984418	KY984467
*Stagonospora paludosa*	S 601 ^NT^ = CBS 135088	–	KF251760	KF251257	KF252262	KF253207
*S. perfecta*	KT 1726A = MAFF 239609	AB797289	AB807579	AB809642	–	AB808555
*S. pseudoperfecta*	KT 889 = CBS 120,236 = MAFF 239607 ^HT^	AB797287	AB807577	AB809641	–	AB808553
*S. tainanensis*	KT 1866 = MAFF 243860	AB797290	AB807580	AB809643	–	AB808556

“–”, sequence is unavailable; Strain with ET (epitype), HT (holotype), NT (neotype), and PT (paratype); Abbreviations: **CBS:** Central Bureau voor Schimmel cultures, Utrecht, The Netherlands; **CGMCC:** China General Microbiological Culture Collection Center; **CPC:** Collection of Pedro Crous housed at CBS; **HHAUF:** Herbarium of Henan Agricultural University: Fungi; **HJAUP:** Herbarium of Jiangxi Agricultural University, Plant Pathology; **JCM:** Japan Collection of Microorganisms**; MFLUCC:** Mae Fah Luang University Culture Collection, Chiang Rai, Thailand; **UESTCC:** University of Electronic Science and Technology Culture Collection, Chengdu, China; **ITS**: internal transcribed spacer; **SSU**: small subunit ribosomal; **LSU**: large subunit ribosomal; ***TEF1***: transcriptional enhancer factor 1-alpha; ***RPB2***: second largest subunit of RNA polymerase II; others are not registered abbreviations.

**Table 2:** Synopsis of morphological characteristics, sequence data, host and locality compared across accepted *Helminthosporium* species.

Species	Conidiophores (μm)	Conida				Sequence Data	Host/Locality	References
		Shape	Colour	Size (µm)	Septa			
*Helminthosporium acaciae*	140–280 × 7–11	Obclavate	Subhyaline to pale brown	31–49 × 10–14	3–6	Absent	On dead branches of *Acacia farnesiana*/Sierra Leone	[7]
*H. ahmadii*	220–650 × 12–15	Obclavate, sometimes rostrate	Brown to dark brown	95–150 × 25–30	5–15	Absent	On dead branches of *Quercus* sp./Pakistan	[7]
*H. aquaticum*	410–580 × 13–17	Obclavate	Pale brown to brown	70–80 × 16–18	8–10	Present	On submerged decaying wood/China	[23]
*H. austriacum*	275–700(–920) × 11.5–19	Obpyriform to lageniform	Pale brown,	(30–)35–48(–97) × (10.0–)13.7–16.5(–19.8)	(4–)5–7(–10)	Present	On dead corticated twigs of *Fagus sylvatica*/Austria	[24]
*H. bambusicola*	55–247 × 4–6	Obclavate	Pale brown, paler towards the apex	36–66 × 6–11	5–8	Absent	On dead *Bambusa* sp. culm/China	[16]
*H. bauhiniae*	350–1100 × 10–15	Obclavate, rostrate	Subhyaline to pale brown	55–145 × 16–18	7–18	Absent	On dead twigs of *Bauhinia tomentosa*/Sierra Leone	[7]
*H. belgaumense*	260–455 × 6.6–10	Ellipsoidal to subspherical	Brown to dark brown	18–24 × 14.9–18.2	1	Absent	On dead twigs of unidentified plant/India	[59]
*H.* *caespitosum*	(21–)27–37(–44) × (11.2–)12.2–14.5(–16.5)	Medium to dark reddish brown, paler toward the apex	Broadly ellipsoid to obclavate, sometimes rostrate	(67–)82–109(–119) × (22.0–)27.3–35.5(–40.5)	(3–)6–10	Present	On dead corticated branches of *Betula* sp./Canada	[24]
*H. chengduense*	133–391 × 7–15	Obclavate, sigmoid, lunate or uncinate	Grey-white to pale brown	41–251 × 8–13	3–16	Present	On decaying branch of unidentified host/China	[30]
*H. chiangraiense*	168–304.5 × 5.5–12	Obclavate, rostrate	Pale brown	141–207 × 14–22	9–13	Present	On dead twigs of unidentified plant/Thailand	[29]
*H. chinense*	214–461 × 6–16	Obclavate	Pale gray to brown	42–109 × 5–11	4–10	Present	On decaying branch of palm trees/China	[30]
*H. chlorophorae*	120–270 × 7–10	Obclavate	Subhyaline to pale brown	52–102 × 8–11	6–9	Present	On dead twigs of *Chlorophora regia*/Sierra Leone	[7]
*H. claviphorum*	(200–)350–900 × 8.5–11	Obclavate	Pale yellowish brown	45–95 × 12–15	5–14	Absent	On rotten branch/Peru	[32]
*H. conidiophorellum*	60–280 × 7–8.5	Subulate	Pale brown	100–147.5 × 9.5–11	11–17	Absent	On dead branches of an unidentified tree/China	[18]
*H. constrictum*	88–205 × 5–8	Obclavate	Pale brown, paler towards the apex	57–120 × 9–12	9–15	Absent	On dead branches of *Trachycarpus fortunei*/China	[15]
*H. cubense*	(25–)50–150 × 4–5	Obclavate or cylindrical	Brown, paler towards the apex	18–62.5 × 6–18	3–7	Absent	On rachis of *Roystonea regia*/Cuba	[60]
*H. dalbergiae*	300–1300 × 10–12(–15)	Obclavate	Straw-coloured to pale brown	58–125 × 12–14	5–17	Present	On dead branches of *Dalbergia sissoo/*Pakistan	[7]
*H. dongxingense*	340–650 × 16–25	Ovoid or obpyriform	Middle brown to brown, paler towards the apex	50–78 × 17–25	6–10	Absent	On dead branches of *Rhododendron* sp./China	[19]
*H. endiandrae*	200–300 × 5–7	Obclavate	Brown	(35–)37–45(–57) × (7–)8(–9)	3(–4)	Present	On leaves of *Endiandra introrsa*/Australia	[24,61]
*H. erythrinicola*	500–1200 × 6–10	Obclavate	Medium brown	(70–)80–90(–110) × (9–)10–11(–12)	(6–)7–8(–12)	Present	On leaves of *Erythrina humeana*/South Africa	[26]
*H.* *genistae*	(155–)280–460(–560) × 15–23	Obclavate to rostrate	Pale golden brown to brown	(41–)51–73(–93) × (10.5–)12.7–15.8(–17.5)	5–12	Present	On dead corticated twigs of *Cytisus scoparius*/France	[24]
*H. guangxiense*	330–850 × 15–20	Obclavate	Middle brown, paler towards the apex	76–110 × 16–22	9–17	Absent	On dead branches of an unidentified tree/China	[18]
*H. guanshanense*	282.5–977.5 × 15–20	Obclavate, rostrate	Pale brown	55–107.5 × 10–15	3–14	Present	On dead branches of an unidentified broadleaf tree/China	This study
*H. hispanicum*	130–540 × 13–22.5	Obclavate	Pale brown	69–99(–130) × (17–)18–21(–24)	(4–)6–11(–14)	Present	On dead corticated twigs of *Juglans regia*/Spain	[24]
*H. hunanense*	70–226 × 5–7	Obclavate	Middle brown, paler towards the apex	56–127 × 10–14	4–12	Absent	On dead branches of an unidentified tree/China	[16]
*H. italicum*	(190–)330–600 × (12–)16–18(–20)	Obclavate	Pale brown to brown, with apical cell paler than other cells,	58–78 × 15–19(–23)	6–11	Present	On dead branch of *Alnus glutinosa*/Italy	[27]
*H. jiulianshanense*	(290–)531–712 × 10–15	Obclavate, rostrate	Pale brown to brown	(57–)78–120 × 13–17.5	6–13	Present	On dead branches of an unidentified broadleaf tree/China	This study
*H. juglandinum*	(175–)215–325(–455) × 11–23	Obclavate, rostrate	Pale brown	(69–)89–145(–205) × (15.0–)16.5–20.0(–25.0)	(5–)9–17(–20)	Present	On dead corticated twigs of *Juglans regia*/Austria	[24]
*H. juglandis*	619–1030 × 10.5–14	Clavate	Brown	50–119 × 10–12.7	4–15	Absent	On living branches of *Juglans regia*/China	[20]
*H. kakamegense*	250–550 × 8–12	Obclavate, rostrate	Subhyaline	30–90 × 8–10	4–15	Absent	On dead attached twig of *Uvariopsis congensis*/Kenya	[10]
*H. kalakadense*	1000–2000 × 17–25	Obclavate	–	45–60 × 13–16	8	Absent	On dead unidentified twig/India	[24,62]
*H. kalopanacis*	63.1–207.5 × 8.3–13.3	Subcylindrical	Pale dark brown	33.2–59.8 × 10–16.6	2–5	Absent	On dead wood of *Kalopanax septemlobus*/Russia	[63]
*H. leucadendri*	100–300 × 4–6(–7)	Obclavate to subcylindrical	Medium brown	(35–)70–110(–170) × (6–)7–8(–11)	(3–)4–6(–10)	Present	On leaves of *Leucadendron* sp./South Africa	[24,64]
*H. ligustri*	127–700 × 9.5–18	Obclavate, rostrate or pseudorostrate	Pale brown, subhyaline towards the apex	24–38.5 × 9.5–13	4–6	Absent	On dead branches of an unidentified tree/China	[18]
*H. livistonae*	Up to 500 × 4–6	subcylindrical	Medium brown	(25–)40–55(–65) × (7–)8–9	(3–)4–6(–7)	Present	On leaves of *Livistona australis*/Australia	[25]
*H. longisinuatum*	20–75 × 3.5–5	Narrowly obclavate	Middle brown, paler toward the apex	65–220(–1000) × 8–10.5	9–22	Absent	On rotten trunk of Palmae/Peru	[32]
*H. magnisporum*	150–270 × 8.5–13.5	Obclavate, rostrate	Pale olive-brown to pale brown, paler toward the apex	100–203 × 12.5–22.5	7–18	Present	On dead fallen branches of an unknown woody plant/Japan	[17,33]
*H. massarinum*	380–810 × 7–9	Obclavate, rostrate	Pale brown	17–56.5 × 5–9	1–8	Present	On vines of *Berchemia racemosa*/Japan	[22]
*H. matsushimae*	20–65 × 3–4.5	Cylindrical	Medium to dark brown	(20–) 50–100 × 6–8.5	(3–) 6–14	Absent	On rotten petiole of Palmae/Peru	[13,32]
*H. mauritianum*	250–750 × 14–20	Obclavate	Subhyaline to rather pale brown	27–55 × 8–13	3–7	Absent	On twigs and stems/Mauritius	[7,65]
*H. meilingense*	544–712.5 × 12.5–17	Obclavate, rostrate	Pale brown	(20.7–)41.5–82.8 × 6.9–10.4	6–13	Present	On dead branches of an unidentified broadleaf tree/China	This study
*H. microsorum*	100–550 × 8–14	Obclavate	Pale to mid golden-brown	60–160 × 12–22	9–17	Present	On twigs of *Quercus ilex*/Italy	[6,66]
*H. multiseptatum*	390–650 × 10–14	Thinly obclavate or nearly whip-like	Pale brown, paler towards the apex	78–190 × 11–16	13–25	Absent	On dead branches of an unidentified tree/China	[15]
*H. nabanhense*	365–557 × 6.5–13.5	Obclavate, rostrate	Pale brown to brown	26.5–46.5 × 6.5–10	3–6	Present	On dead branches of an unidentified broadleaf tree/China	[31]
*H. nanjingense*	250–470 × 6.9–7.7	Subulate or nearly whip-like	Pale brown	64.5–170.5 × 7.3–10.3	6–17	Present	On dead branches of an unidentified tree/China	[21]
*H. novae-zelandiae*	165–330 × 12.5–14.5	Obclavate to fusiform, sometimes shortly rostrate	Golden brown to dark brown, paler toward the apex	56–103 × 16–21.5	(5–)6–7(–8)	Absent	On dead wood and bark of *Vitex lucens*/New Zealand	[67]
*H. obpyriforme*	225–460 × 9.5–13	Obpyriform	Middle brown, paler towards the apex	47–74 × 14–19	5–9	Absent	On dead branches of an unidentified tree/China	[18]
*H. oligosporum*	(17–)22–35(–46) × (8.0–)8.5–10.5(–11.5)	Obclavate, sometimes rostrate, smooth but occasionally wrinkled with age	Pale brown to brown, paler toward the apex	(37–)59–80(–124) × (14.8–)15.8–18.0(–20.0)	6–12(–16)	Present	On dead corticated twigs of *Tilia cordata*/Austria	[24]
*H. ovoideum*	380–510 × 15–25	Ovoid to ellipsoidal	Moderately brown, paler towards the apex	27–61 × 13–21	3–8	Absent	On dead branches of an unidentified tree/China	[18]
*H. palmigenum*	70–180(–250) × 7–10	Obclavate	Below pale brown, upper subhyaline	27–47 × 6.5–9	4–9	Absent	On rotten fruit of *Cocos nucifera*/Papua New Guinea	[68]
*H. pseudomicrosorum*	155–288 × 11–15	Obclavate	Brown, paler towards the apex	82–142 × 17–27	7–16	Absent	On dead branches of an unidentified tree/China	[18]
*H. quercicola*	(115–)133–226(–300) × 14–20	Obclavate	Brown	60–100 × 15–22	8–10	Absent	On dead corticated branches of *Quercus* cf. *reticulata*/USA	[24]
*H. quercinum*	(40–)74–199(–332) × 11–18	Obclavate, rostrate	Brown	(47–)78–130(–201) × (13.2–)15.3–18.0(–20.5)	8–13(–20)	Present	On dead corticated twigs of *Quercus petraea*/Austria	[24]
*H. sichuanense*	300–550 × 14–25	Obclavate,	Middle brown, paler towards the apex	41–86 × 10–14	5–11	Absent	On dead branches of an unidentified plant/China	[14]
*H. sinense*	220–370 × 6–8.5	Obclavate	Pale brown	37–60 × 5.5–8.5	2–7	Present	On dead branches of an unidentified broadleaf tree/China	[31]
*H. solani*	120–600 × 9–15	Obclavate	Subhyaline to brown	24–85 × 7–11	2–8	Present	On stem of *Solanum nigrum*/England	[7,49]
*H. spurirostrum*	200–600 × 18–23	Obclavate, sometimes pseudorostrate	Moderately brown to brown, paler to the apex	27–73 × 7–15.5	4–7	Absent	On dead branches of an unidentified plant/China	[14]
*H. subhyalinum*	120–200 × 6–8.5	Thinly obclavate	Subhyaline	72–125 × 9–11.5	6–9	Absent	On living leaves of *Phoenix hanceana*/China	[15]
*H. submersum*	239–423 × 8.5–15.5	Obclavate, rostrate	Pale brown to mid-brown	41–55 × 14.5–18.5	6–10	Present	On submerged decaying wood/China	[28]
*H. syzygii*	150–400 × 10–15	Obclavate	Medium brown	(70–)80–100(–150) × (19–)22–23(–25)	(7–)9–12	Present	On bark canker of *Syzygium* sp./South Africa	[26]
*H.* *tiliae*	(68–)79–133(–150) × 9–15	Obclavate to rostrate	Pale to golden brown	(57–)74–111(–122) × (13.5–)13.7–19.0(–24.5)	7–18(–25)	Present	On dead corticated branches of *Tilia platyphyllos*/Austria	[24]
*H. velutinum*	(163–)340–698(–960) × 14–26	Obclavate to rostrate	Pale golden brown to brown	(42–)56–89(–142) × (11–)14.3–18.5(–24.7)	6–18	Present	On dead corticated twigs of *Fagus sylvatica*/Austria	[4,24]
*H. yunnanense*	560–680 × 12.5–15.5	Obclavate, sigmoid, lunate or uncinate	Pale brown	30.5–55.5 × 9–11	4–7	Present	On dead branches of an unidentified broadleaf tree/China	[31]

All conidia are smooth, except for *H. conidiophorellum*, *H. endiandrae* and *H. oligosporum*, which are verrucose or roughened; All conidiogenous cells are polytretic except for *H. caespitosum*, *H. endiandrae*, *H. leucadendri* and *H. oligosporum*, which are monotretic; All species are reported from terrestrial habitats except for *H. aquaticum* and *H. submersum*, which are recorded in aquatic habitats; All conidia are solitary except for *H. endiandrae*, *H. massarinum* and *H. sinense*, which produce catenate conidia.

## Data Availability

All sequences generated in this study were submitted to GenBank.
